# Sepsis-associated microvascular dysfunction measured by peripheral arterial tonometry: an observational study

**DOI:** 10.1186/cc8055

**Published:** 2009-09-25

**Authors:** Joshua S Davis, Tsin W Yeo, Jane H Thomas, Mark McMillan, Christabelle J Darcy, Yvette R McNeil, Allen C Cheng, David S Celermajer, Dianne P Stephens, Nicholas M Anstey

**Affiliations:** 1 International Health Division, Menzies School of Health Research and Charles Darwin University, Rocklands Drive, Darwin, NT 0810, Australia; 2 Division of Medicine, Royal Darwin Hospital, Rocklands Drive, Darwin, NT 0810, Australia; 3 Intensive Care Unit, Royal Darwin Hospital, Rocklands Drive, Darwin, NT 0810, Australia; 4 Department of Medicine, University of Sydney and Department of Cardiology, Royal Prince Alfred Hospital, Missenden Road, Sydney, NSW 2006, Australia

## Abstract

**Introduction:**

Sepsis has a high mortality despite advances in management. Microcirculatory and endothelial dysfunction contribute to organ failure, and better tools are needed to assess microcirculatory responses to adjunctive therapies. We hypothesised that peripheral arterial tonometry (PAT), a novel user-independent measure of endothelium-dependent microvascular reactivity, would be impaired in proportion to sepsis severity and related to endothelial activation and plasma arginine concentrations.

**Methods:**

Observational cohort study in a 350-bed teaching hospital in tropical Australia. Bedside microvascular reactivity was measured in 85 adults with sepsis and 45 controls at baseline and 2-4 days later by peripheral arterial tonometry. Microvascular reactivity was related to measures of disease severity, plasma concentrations of L-arginine (the substrate for nitric oxide synthase), and biomarkers of endothelial activation.

**Results:**

Baseline reactive hyperaemia index (RH-PAT index), measuring endothelium-dependent microvascular reactivity; (mean [95% CI]) was lowest in severe sepsis (1.57 [1.43-1.70]), intermediate in sepsis without organ failure (1.85 [1.67-2.03]) and highest in controls (2.05 [1.91-2.19]); *P *< 0.00001. Independent predictors of baseline RH-PAT index in sepsis were APACHE II score and mean arterial pressure, but not plasma L-arginine or markers of endothelial activation. Low baseline RH-PAT index was significantly correlated with an increase in SOFA score over the first 2-4 days (r = -0.37, *P *= 0.02).

**Conclusions:**

Endothelium-dependent microvascular reactivity is impaired in proportion to sepsis severity and suggests decreased endothelial nitric oxide bioavailability in sepsis. Peripheral arterial tonometry may have a role as a user-independent method of monitoring responses to novel adjunctive therapies targeting endothelial dysfunction in sepsis.

## Introduction

Mortality from severe sepsis remains high, despite advances in its management [1]. Organ failure commonly occurs despite the achievement of normal haemodynamics in response to fluid resuscitation, vasopressors and the treatment of infection. This may be due to impaired vasomotor regulation of the microcirculation [2]. In sepsis, the endothelium has key roles in regulating vascular tone and permeability and its activation is pivotal in initiating both the inflammatory and coagulation cascades [3].

Endothelial function is assessed clinically by the ability of blood vessels to vasodilate in response to pharmacological stimuli or to shear stress, and is primarily dependent on endothelial nitric oxide (NO) production [4]. As a result, many clinical studies investigating the endothelium in sepsis have measured circulating endothelial activation markers, as a surrogate for endothelial function. Current techniques for measurement of endothelial function, such as laser Doppler, plethysmography and flow-mediated dilatation of the brachial artery, require skilled operators and are technically difficult to perform at the bedside. Some studies have assessed endothelial function by measuring reactive hyperaemia in human sepsis using these operator-dependant techniques [5-10]. These studies have generally shown normal baseline blood flow and impaired reactive hyperaemic responses in sepsis, but have been small (n = 8 to 45) and have not correlated reactive hyperaemia with L-arginine or circulating markers of endothelial activation. More recently, investigators using dynamic near-infrared spectroscopy (NIRS) have found impaired microvascular responses in sepsis; however, the nature of the relation between NIRS and endothelial NO activity is unclear [11].

**Figure 1:**
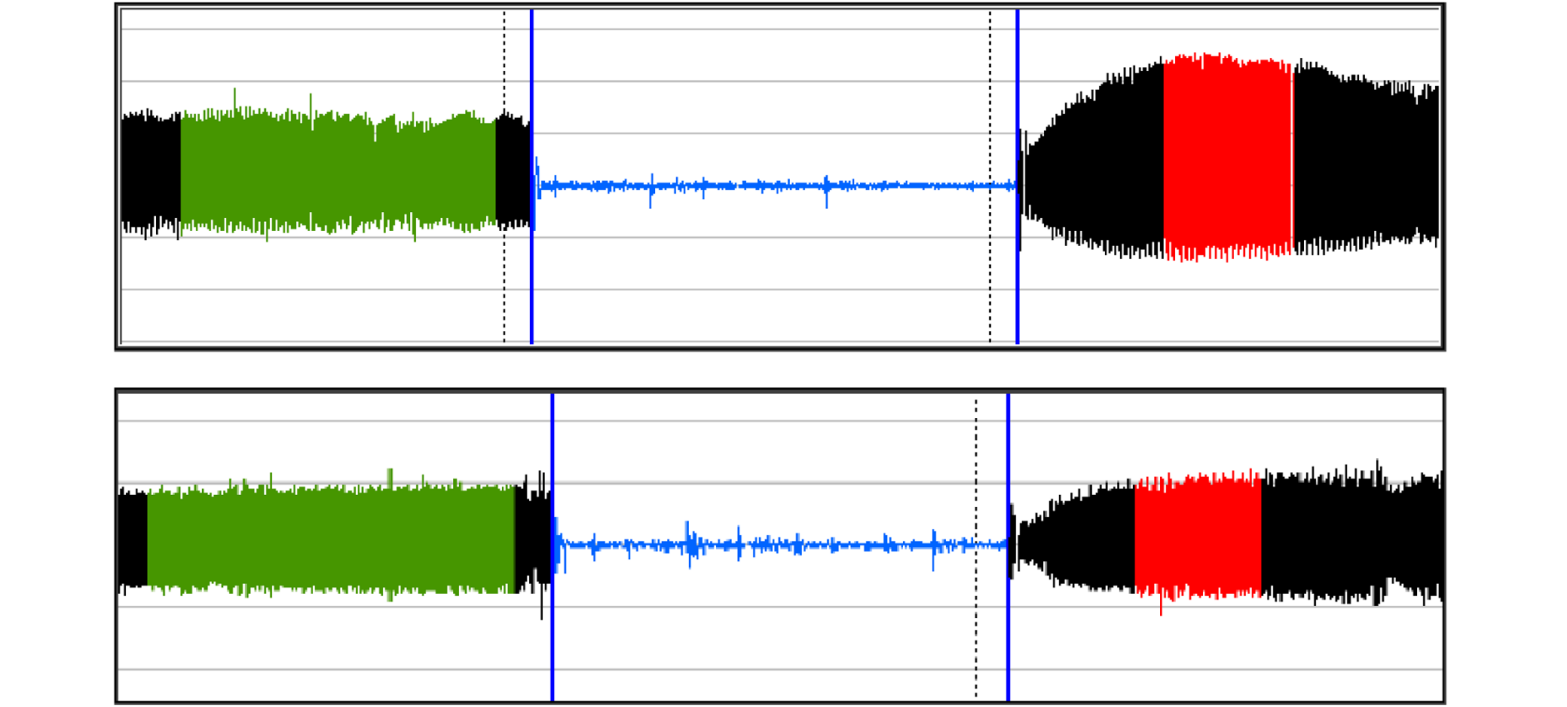
Representative normal and abnormal peripheral arterial tonometry traces.  The tracings represent the pulse wave amplitude from a fingertip over a 15-minute period. The y axis is pulse wave amplitude in arbitrary units (derived from millivolts). The top trace was taken from a control subject whose reactive hyperaemia peripheral arterial tonometry; (RH-PAT) index was 1.98, and the bottom from a severe sepsis subject whose RH-PAT index was 1.16. The horizontal axis is time. The first shaded section is averaged as a baseline signal. The middle section is arterial occlusion, with consequent loss of the pulse wave signal. The final section is the pulse wave amplitude following release of the cuff. The random vertical spikes are movement artefacts. In the top trace there is reactive hyperaemia, with an increase in average pulse wave amplitude. The shaded post-occlusion section is compared with the shaded baseline section to give a ratio -- the RH-PAT index.

Reactive hyperaemia peripheral arterial tonometry (RH-PAT) is a novel, simple and user-independent bedside technique used to measure microvascular endothelial function [12] (Figure 1). It is increasingly being used to measure endothelial function as a cardiovascular risk assessment tool in ambulatory patients [12-16], including in the third-generation Framingham Heart Study cohort [17]. RH-PAT has been shown to be at least 50% dependent on endothelial NO activity [18]. RH-PAT uses finger probes to measure digital pulse wave amplitude detected by a pressure transducer, and has been validated against the operator-dependent flow-mediated dilatation method [19,20] and with endothelial function in other vascular beds, including the coronary arteries [13]. Using RH-PAT, we have demonstrated endothelial dysfunction in subjects with severe malaria [21] but it has not previously been evaluated in subjects with sepsis.

Vasodilatory shock in sepsis has been hypothesized to reflect a state of NO excess. However, several recent isotope studies have shown no net increase in NO synthesis in humans with sepsis [22-24]. To explain this, it has been proposed that sepsis may be a state of imbalance between the NOS isoforms inducible NOS and endothelial NOS in the microvasculature [25]. This could lead to a relative deficiency of endothelial NO, which is required to maintain the microvascular endothelium in a healthy, quiescent state.

Another possible reason for endothelial NO deficiency is decreased availability of L-arginine, the substrate for NOS and the precursor for NO [26]. Sepsis has been hypothesised to be an arginine-deficient state [27], although plasma L-arginine levels in humans with sepsis have been variably reported to be high [28], normal [29,30] or low [22,31,32]. Decreased plasma L-arginine has been linked to decreased NO production in animal and *in vitro *models [33].

We hypothesised that RH-PAT would be a feasible technique to measure microvascular reactivity in sepsis and that microvascular reactivity would be impaired in subjects with sepsis in proportion to disease severity. Our secondary hypotheses were that microvascular reactivity would correlate with plasma L-arginine and measures of endothelial activation, and that plasma L-arginine concentrations would be decreased in sepsis.

## Materials and methods

### Study design and setting

We performed a prospective observational cohort study in a 350-bed teaching hospital in tropical northern Australia, with an 18-bed mixed intensive care unit (ICU). Approval was obtained from Human Research Ethics Committee of the Menzies School of Health Research and the Department of Health and Community Services, Darwin. Written informed consent was obtained from all participants or next of kin.

### Participants

Between March 2006 and November 2007, all adult subjects (≥ 18 years) admitted to the hospital were screened regarding eligibility for the study. Inclusion criteria for sepsis subjects were: suspected or proven infection; presence of two or more criteria for the systemic inflammatory response syndrome within the past four hours [34]; and admission to ICU within the preceding 24 hours or to the wards within the preceding 36 hours. Exclusion criteria were coagulopathy (platelets ≤ 20 × 10^9^/L, activated partial thromboplastin time ≥ 70 seconds, international normalized ratio ≥ 2.0); smoking of tobacco within the preceding four hours; and current administration of intravenous nitrates. Control subjects were recruited from hospital patients with no clinical or laboratory evidence of inflammation or infection, and who had not met systemic inflammatory response syndrome criteria within the preceding 30 days. Severe sepsis was defined as sepsis with organ dysfunction or shock at the time of enrolment according to American College of Chest Physicians/Society of Critical Care Medicine consensus criteria [34,35].

### Measurement of microvascular reactivity

Sepsis subjects underwent standardised demographic and clinical data collection, bedside RH-PAT measurement (Endopat 2000, Itamar Medical, Caesarea, Israel), and blood collection at days 0 and 2 to 4. All studies were performed after resuscitation and at least one hour of hemodynamic stability (defined as no change in vasopressor dose or need for fluid boluses) in a quiet room at 25°C, with the patient recumbent. Control subjects had the same assessment at a single time point.

In this study, probes were placed on the index fingers of both hands of all patients, or on other fingers if the index fingers were not suitable. Digital pulse wave amplitude was recorded from both hands for a resting baseline period of five minutes and then a blood pressure cuff was rapidly inflated on the study arm up to 200 mmHg, or 50 mmHg above systolic blood pressure, whichever was greater. After five minutes ± 10 seconds, the cuff was deflated. Pulse wave amplitude was then recorded for a further five minutes. An automated computerised algorithm provided by the manufacturer (Endo-PAT 2000 software version 3.1.2, Itamar Medical, Caesarea, Israel) was used to calculate a post occlusion-pre occlusion ratio (RH-PAT index), thus making the measurements user independent. The software also normalises the RH-PAT index to the control arm to correct for changes in systemic vascular tone (Figure 1).

There was no systematic difference between RH-PAT indices generated by different observers. We have previously examined the reproducibility of RH-PAT measurements by repeating them after 0.5 to 0.75 hours in 37 healthy adults [21]. Reproducibility was acceptable according to the method of Bland and Altman [36], and was comparable with previous reproducibility results for RH-PAT [37] and with those obtained with the flow-mediated dilatation method [38].

### Laboratory assays

Blood was collected in lithium heparin tubes at each time point and the plasma was frozen. Plasma arginine concentrations were determined using high-performance liquid chromatography, with a method modified from van Wandelen and Cohen [39]. To assess circulating measures of endothelial activation, intra-cellular adhesion molecule-1 (ICAM1) and E-selectin were measured by ELISA (R&D Systems, Minneapolis, Minnestoa, USA). Plasma IL-6 was measured by flow cytometry using a cytokine bead array (BD Biosciences, San Jose, California, USA). *Ex vivo *plasma arginase activity causes significant degradation of L-arginine at room temperature [40], thus only L-arginine levels derived from blood frozen within 30 minutes of collection were included in the analysis.

### Statistical methods

Predefined groups for analysis were sepsis without organ failure, severe sepsis and controls. Continuous variables were compared using Student's t-test and analysis of variance or Mann Whitney U test for parametric and non-parametric variables, respectively. Categorical variables were compared using Fisher's exact test. Correlates with baseline RH-PAT index were determined using Pearson's (parametric) or Spearman's (non-parametric) coefficient for univariate analysis. For multivariate analysis, linear regression with backward selection was used. To examine longitudinal correlations, linear mixed-effects models were used. A two-sided *P *value of < 0.05 was considered significant. All analyses were performed using Stata version 10 (Stata Corp, College Station, Texas, USA).

## Results

### Participants

Over the 19-month study period, 85 subjects with sepsis and 45 control subjects were enrolled. Of the sepsis subjects, 54 had organ failure due to sepsis at baseline (severe sepsis group) and 31 did not (sepsis without organ failure). The three groups were well matched in terms of risk factors for endothelial dysfunction and other baseline characteristics (Table 1). Of the 85 sepsis subjects, 92% had community-acquired sepsis, with no preceding trauma or surgery, and pneumonia was the most common focus of infection.

**Table 1:** Baseline characteristics of participants

	Severe sepsis	Sepsis without organ failure	Control	*P *value^a^
**N**	54	31	45	
**Age^b^**	52.4 (48.3-56.5)	50.8 (46.5-55.2)	47.2 (43.1-51.4)	NS
**Male n (%)**	33 (61)	21 (68)	30 (67)	NS
**Diabetic n (%)**	18 (33)	7 (23)	14 (31)	NS
**Smoker n (%)**	28 (57)	12 (39)	18 (41)	NS
**IHD n (%)**	9 (17)	6 (19)	6 (13)	NS
**On statin n (%)**	13 (24)	9 (29)	13 (29)	NS
**APACHE II^c^**	19.0 (15-23)	7.5 (5-11)		< 0.0001
**SOFA score^c^**	6 (3-9)	1 (0-2)		< 0.0001
***Focus of infection -- n (%)***				
**Pleuropulmonary n (%)**	26 (48)	16 (52)		
**Skin/soft tissue n (%)**	9 (17)	9 (29)		
**Intra-abdominal n (%)**	6 (11)	1 (3)		
**Urinary n (%)**	4 (7)	3 (10)		
**Other n (%)**	9 (17)	2(6)		
***Causative organism***				
**None cultured n (%)**	25 (46)	20 (65)		
**Gram positive bacterium n (%)**	15 (28)	5 (16)		
**Gram negative bacterium n (%)**	14 (26)	6 (19)		
***Origin of sepsis***				
**Community-acquired n (%)**	47 (87)	30 (97)		
**Nosocomial n (%)**	7 (13)	1 (3)		

a. For difference between all three groups by one way analysis of variance

b. Mean (95% confidence interval)

c. Median (interquartile range)

APACHE II = Acute Physiology and Chronic Health Evaluation II; IHD = ischaemic heart disease; NS = not significant; SOFA = Sequential Organ Failure Assessment score

### Baseline microvascular reactivity

**Figure 2:**
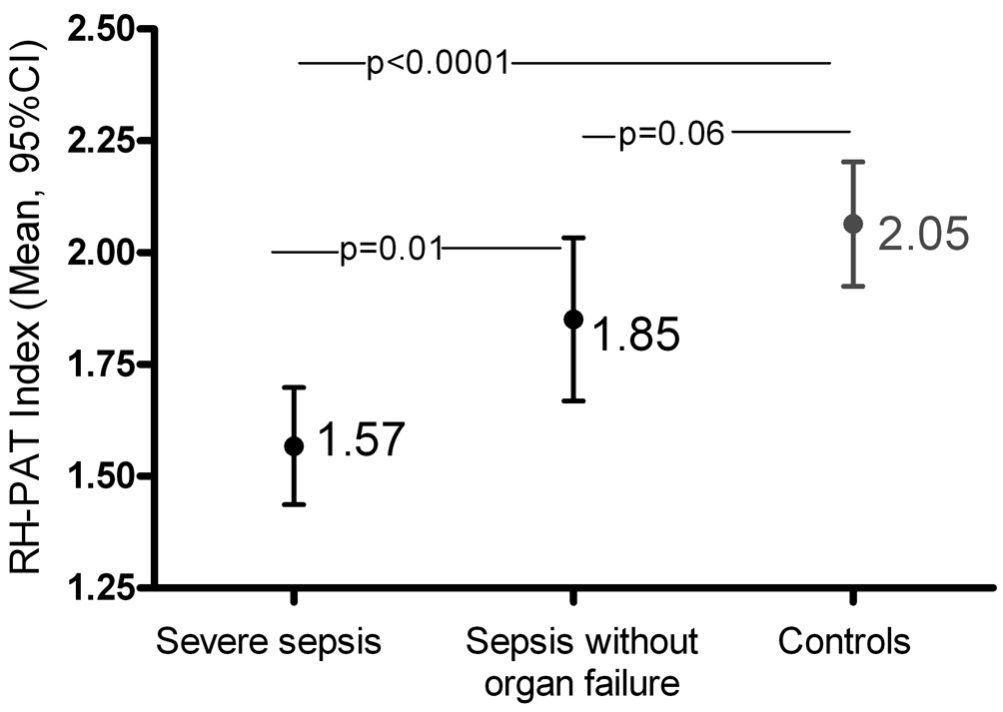
Baseline microvascular reactivity is impaired in sepsis, in proportion to disease severity.  Solid circles represent mean values, with error bars representing 95% confidence intervals (CI). *P *values indicate pairwise comparisons between groups. RH-PAT = reactive hyperaemia peripheral arterial tonometry.

Baseline microvascular reactivity was impaired in sepsis subjects compared with controls (*P *< 0.0001; Table 2). Mean RH-PAT index was lowest in the severe sepsis group (1.57, 95% confidence interval (CI): 1.43 to 1.70), intermediate in the sepsis without organ failure group (1.85, 95% CI: 1.67 to 2.03), and highest in the control group (2.05, 95% CI: 1.91 to 2.19; *P *< 0.00001; Figure 2). Subjects with severe sepsis were more likely to have endothelial dysfunction than control subjects (odds ratio (OR) 9.4, 95% CI: 3.5 to 25.0). This relation persisted after controlling for known associations with and risk factors for endothelial dysfunction (diabetes, smoking, ischaemic heart disease, chronic renal disease, hypercholesterolaemia, hypertension, statin use and age; adjusted OR 17.0, 95% CI: 5.0 to 58.0). Within the severe sepsis group, mean RH-PAT index was not significantly different in the 27 subjects requiring vasopressors (1.48, 95% CI: 1.30 to 1.66) than in those not requiring vasopressors (1.64, 95% CI: 1.39 to 1.89; *P *= not significant (NS)). In those receiving noradrenaline (n = 25), there was no correlation between RH-PAT index and noadrenaline dose (r = 0.19, *P *= NS). There was also no relation between body temperature and RH-PAT index. Males (1.76, 95% CI: 1.62 to 1.89) had higher baseline microvascular reactivity than females (1.50, 95% CI: 1.32 to 1.68; *P *= 0.02).

**Table 2:** RH-PAT index and related variables at time of initial measurement

	Severe sepsis	Sepsis without organ failure	Control	*P *value pooled sepsis v control	*P *value severe sepsis vs SWOF
**N**	54	31	45		
**RH-PAT index^a^**	1.57 (1.43-1.70)	1.85 (1.67-2.03)	2.05 (1.91-2.19)	< 0.00001	0.01
**Plasma L-arginine (μmol/L)**	35.8 (30.2-41.4)	40.9 (33.5-48.3)	80.4 (72.3-88.6)	< 0.00001	NS
**MAP (mmHg)^a^**	77 (74-81)	89 (83-95)	83 (79-87)	NS	0.0006
**Receiving vasopressors n (%)**	27 (50)	0			
**Noradrenaline dose (μg/kg/min)^b, c^**	0.08 (0.03-0.42)				
**Receiving assisted ventilation n (%)**	20 (37)	0			
**CVP (cmH20)^a^**	12.2 (10.3-14.1)				
**Plasma ICAM-1 (ng/ml)^b^**	811 (500-1502)	507 (368-673)	323 (252-397)	< 0.00001	0.003
**Plasma E-selectin (ng/ml)^b^**	329 (138-502)	90 (51-164)	38 (26-63)	< 0.00001	0.0003
**Plasma IL 6 (pg/ml)^b^**	385 (124-996)	148 (46-315)	5 (2-8)	< 0.00001	0.009
**White blood cell count^a^**	16.7 (14.2-19.2)	15.5 (13.3-17.7)	8.4 (6.9-9.8)	< 0.00001	NS
**C-reactive protein^b^**	190 (131-255)	102 (84-234)	7 (3-24)	< 0.00001	NS

a. mean (95% confidence interval)

b. Median (interquartile range)

c. Of 27 patients receiving vasopressors, 25 were receiving noradrenaline

d. Severe sepsis n = 30, sepsis without organ failure n = 26, control n = 2.

CVP = central venous pressure; ICAM = intra-cellular adhesion molecule-1; NS = not significant; MAP = mean arterial pressure; RH-PAT = reactive hyperaemia peripheral arterial tonometry; SWOF = sepsis without organ failure.

RH-PAT was well tolerated by all subjects. In 18 of 227 measurements (8%), a result was not obtainable. This occurred in 15 of 182 measurements (8%) in sepsis subjects and 3 of 45 (7%) in controls and was due either to inability to obtain a baseline pulse wave reading, or failure to completely occlude forearm blood flow due to oedema.

Plasma markers of endothelial activation (ICAM-1 and E-selectin) were both significantly raised in sepsis subjects compared with controls (Table 2); however, they did not correlate with RH-PAT index. Blood lactate levels were routinely measured only in subjects with severe sepsis, in whom the baseline median lactate was 1.6 mmol/L (range 0.5 to 12.7; interquartile range (IQR) 1.0 to 2.3). Among severe sepsis subjects, lactate correlated inversely with RH-PAT index, but this was not statistically significant (r = -0.28, *P *= 0.06).

Among all sepsis subjects, baseline RH-PAT index correlated with mean arterial pressure (MAP; r = 0.55, *P *< 0.0001) and serum albumin (r = 0.27, *P *= 0.03), and was inversely related to Acute Physiology and Chronic Health Evaluation (APACHE) II score (r = -0.36, *P *= 0.002), C-reactive protein (r = -0.30, *P *= 0.02) and the cardiovascular component of the Sequential Organ Failure Assessment (SOFA) score (r = -0.29, *P *= 0.01), but not with total SOFA score. Independent predictors of baseline RH-PAT index on multivariate analysis were APACHE II score (β = -0.014, *P *= 0.03) and MAP (β = 0.012, *P *< 0.0001).

### Baseline plasma L-arginine

**Figure 3:**
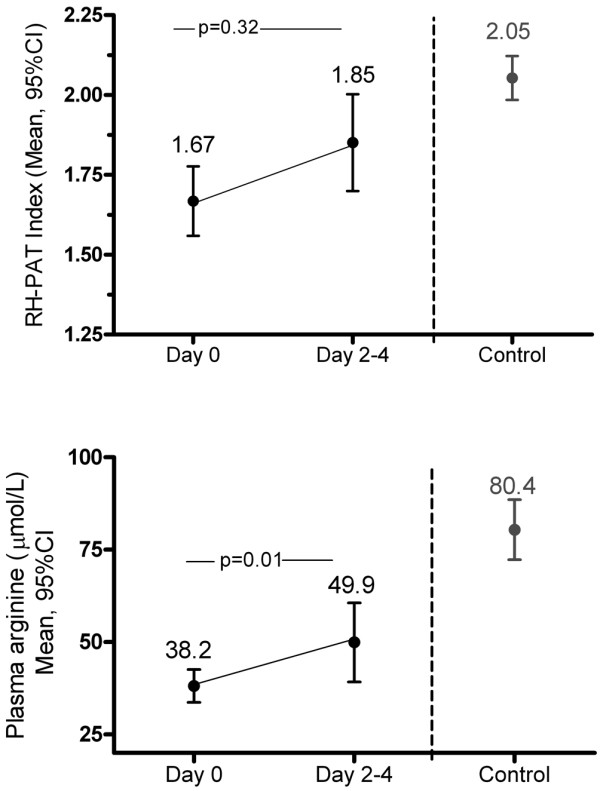
Longitudinal change in microvascular reactivity and plasma arginine in sepsis subjects.  Solid circles represent mean values, with error bars representing 95% confidence intervals (CI). RH-PAT = reactive hyperaemia peripheral arterial tonometry.

In the subjects whose blood samples were processed within 30 minutes of collection, baseline mean plasma L-arginine concentration was significantly lower in sepsis subjects (38.6 μmol/L, 95% CI: 34.2 to 43.1; n = 56) than in controls (80.3 μmol/L, 95% CI: 72.5 to 88.1; n = 27; *P *< 0.0001). There was no significant difference in L-arginine levels between severe sepsis and sepsis without organ failure (Figure 3). When all subjects including controls were considered, baseline plasma L-arginine correlated with baseline RH-PAT index (r = 0.32, *P *= 0.007); however, this association was no longer significant when stratified by disease severity.

### Longitudinal changes in RH-PAT and L-arginine

Longitudinal RH-PAT readings were only available in 70% of subjects. There was no difference in disease severity, as measured by APACHE II score, in those with (median 14, IQR 8 to 23) and without (median 15.5, IQR 8.5 to 20.5; *P *= NS) longitudinal data. In sepsis subjects, there was no statistically significant change in mean RH-PAT index from baseline to day 2 to 4 (95% CI: 1.67 to 1.85, *P *= NS; Figure 3). The same was true in the severe sepsis subgroup (95% CI: 1.57 to 1.76, *P *= NS). In contrast, mean plasma L-arginine concentrations significantly increased from baseline to day 2 to 4 (95% CI: 38.2 to 49.9 μmol/L, *P *= 0.01). In a mixed-effects linear regression model, change in microvascular reactivity over the first 2 to 4 days of treatment correlated significantly with increasing MAP and decreasing C-reactive protein, but not with change in plasma L-arginine.

### Subject outcomes

Low baseline RH-PAT index was significantly correlated with an increase in SOFA score over the first 2 to 4 days (r = -0.37, *P *= 0.02). In subjects whose SOFA score worsened over the first 2 to 4 days, the median RH-PAT index was 1.54, compared with 1.74 in those whose SOFA score improved or did not change (*P *= 0.01). At both hospital discharge and 28-day follow-up, 8 of 85 (9%) subjects with sepsis had died. Among those with septic shock at baseline, 6 of 29 (21%) had died at 28-day follow-up. The mean baseline RH-PAT index was 1.67 among survivors and 1.60 among non-survivors (*P *= NS). The strongest baseline predictors of death on univariate analysis were APACHE II score (*P *= 0.008), SOFA score (*P *= 0.002) and IL-6 level (*P *= 0.004).

## Discussion

To the authors' knowledge, this is the largest published study to date assessing reactive hyperaemia in human sepsis and the first to use peripheral arterial tonometry. We have found that endothelium-dependent microvascular reactivity is impaired in sepsis, in proportion to disease severity, even after controlling for known associations with endothelial dysfunction, suggesting that sepsis itself is the explanation for the observed impairment in microvascular reactivity, rather than traditional cardiovascular risk factors. Furthermore, the degree of impairment of baseline microvascular reactivity predicted subsequent deterioration in organ function.

RH-PAT proved to be a practical and feasible method of measuring microvascular reactivity at the bedside in critically ill septic subjects, with a low proportion of technical failures, which were no more common in sepsis subjects than in controls, and which showed no relation with noradrenaline dose. The findings of this study are generally consistent with those of the previous small studies of reactive hyperaemia in adult subjects with sepsis using other methods, which were generally user-dependant and of limited availability.

Plethysmographic measures of forearm blood flow in sepsis have found a post occlusion-pre occlusion ratio of 1.6 [9] and forearm skin laser Doppler studies have found a ratio of 1.4 [5]. These results are very similar to our observed ratio of 1.57, suggesting that the finding of impaired reactive hyperaemia in adults with sepsis is a true phenomenon, which is independent of the method used to measure it.

Compared with laser Doppler flowmetry, venous plethysmography and flow-mediated dilatation of the brachial artery, PAT requires less staff training and simpler equipment, has less potential for inter-observer variability, and is easier to perform on uncooperative patients. PAT has also been validated with regards to accuracy [13,19,20] and reproducibility [37,41]. Disadvantages of PAT include the expense of disposable finger probes.

Because RH-PAT is at least 50% NO-dependent [18], impaired RH-PAT responses in sepsis suggest reduced endothelial NO bioavailability. Our results are in accord with increasing data from radiolabelled arginine flux studies suggesting that NO synthesis is decreased in sepsis [22-24]. Impaired RH-PAT has been demonstrated to be reversible with L-arginine infusion in malaria caused by *Plasmodium falciparum*, providing direct evidence for NO dependence in acute inflammatory states [21]. However, we cannot exclude contributions by other mechanisms, including impaired production of prostacyclin and endothelium-derived hyperpolarizing factor [42,43].

There was a significant correlation between plasma L-arginine and microvascular reactivity when all subjects were considered together, but this was not significant within groups. Furthermore, the improvement of plasma L-arginine over the first 2 to 4 days was not significantly correlated with change in microvascular reactivity. These findings suggest that NO production and endothelial function in sepsis are influenced by other factors in addition to circulating L-arginine. Such factors may include an increase in competitive inhibitors of NOS, such as asymmetric dimethylarginine [44]; deficiency of NOS cofactors such as tetrahydrobiopterin; NO quenching by microvascular reactive oxygen intermediates [45]; and the enhanced local expression and activity of endothelial cell arginase [46]. The observation of higher microvascular reactivity in males compared with females is an unexpected finding; previous studies have found better microvascular function in females than males, both in non-inflammatory states [47] and in response to infusion of lipopolysaccharide [48]. However, gender-specific microvascular function has not previously been reported in sepsis.

The marked hypoargininaemia, which we found in subjects with sepsis, supports the hypothesis that L-arginine is decreased in sepsis, independent of trauma [27]. This finding is strengthened by the fact that we only included subjects within 24 to 36 hours of admission, with standardised sepsis criteria and with more than 90% having community-acquired sepsis.

Targeting tissue oxygen delivery [49] or the splanchnic microcirculation [50] as resuscitation goals in sepsis have not been shown to improve outcomes. What, then, is the significance of monitoring the microvascular endothelium in sepsis? Endothelial cells have multiple roles in sepsis pathophysiology, including the regulation of microcirculatory vasomotor tone and the regulation of coagulation, immune and inflammatory responses and microvascular barrier function. Preliminary studies aimed at increasing endothelial NO bioavailability in sepsis have shown promising results [51] and the interventions which have been demonstrated to improve outcomes in sepsis (activated protein C [52], early goal directed therapy [53] and intensive insulin therapy [54]) could all potentially be mediated, at least in part, via attenuation of endothelial cell dysfunction [55]. Thus, monitoring of microvascular and endothelial function are likely to be important components of future trials of adjunctive treatments in sepsis.

Our study has several potential limitations. Baseline blood flow measurements were not available, and it is possible that the apparent decrease in reactive hyperaemia in sepsis is an artefact of marked baseline vasodilatation. This could potentially limit the subjects' ability to respond to ischaemia by increased blood flow, because they already have near-maximal vasodilatation. This is unlikely to be the case because baseline forearm blood flow in septic subjects has been found to be normal or decreased by multiple investigators [6,7,10,56]. Furthermore, skeletal muscle has the capacity to increase blood flow by up to 10-fold [57], which greatly exceeds the increase seen in both healthy and septic subjects in this and other studies.

Although we controlled for the major factors influencing endothelial function, we cannot exclude minor influences of altered thyroid or adrenal function. Due to variations in sample processing time, we were unable to determine accurate plasma arginine values for all subjects. Thus the reported arginine values may not be fully representative of the groups as a whole. Of the subjects who had an initial measurement of RH-PAT index, 70% had a repeat measurement 2 to 4 days later. Although those who were not followed up had a similar baseline APACHE II score to those who were followed up, this may not have been a representative population, because subjects who rapidly improved and were discharged home did not have repeat measurements. Thus the observed degree of recovery in microvascular reactivity is likely to be an underestimate.

The mortality rate in this cohort was low (hospital and 28-day mortality 9% overall and 21% among those with septic shock). Although this is consistent with the relatively low mortality rate in severe sepsis previously documented in our ICU [35], it does mean that the study may have been underpowered to detect associations of measured variables with mortality.

## Conclusions

In summary, we have found that peripheral arterial tonometry is a feasible tool for measuring microvascular reactivity in sepsis, and that it is impaired in sepsis in proportion to disease severity, suggesting reduced endothelial function and decreased endothelial NO bioavailability. Baseline RH-PAT was useful in predicting subsequent deterioration in organ dysfunction, although this should be reproduced by other investigators before its clinical utility can be confirmed. Given the growing interest in HMG CoA reductase inhibitors [58] and other potential adjunctive therapies targeting the endothelium in sepsis [55], better tools for monitoring the response of the endothelium in clinical trials are needed. RH-PAT is an attractive option for such studies, as other current methods are user-dependent and have limited availability.

## Key messages

• Current tools for assessing endothelial function in patients with sepsis are generally user dependant and are not widely available.

• Peripheral arterial tonometry, a simple, user-independent technique for measuring endothelium-dependent microvascular reactivity is feasible in patients with sepsis.

• Endothelium-dependent microvascular reactivity is impaired in sepsis, in proportion to disease severity, and may predict subsequent deterioration in organ function.

## Abbreviations

APACHE: Acute Physiology and Chronic Health Evaluation; CI: confidence interval; ELISA: enzyme-linked immunosorbent assay; ICAM-1: intra-cellular adhesion molecule-1; ICU: intensive care unit; IL: interleukin; MAP: mean arterial pressure; NIRS: near infrared spectroscopy; NO: nitric oxide; NOS: nitric oxide synthase; NS: not significant; OR: odds ratio; RH-PAT: reactive hyperaemia peripheral arterial tonometry; SOFA: Sequential Organ Failure Assessment.

## Competing interests

DC has received research support (as equipment) from Itamar Medical, the manufacturer of the RH-PAT device, and has received speaker's fees (less than US$1000 per year) for speaking at Itamar-sponsored educational events. The other authors have no competing interests.

## Authors' contributions

Study design was performed by JSD, NMA, TWY, DPS and DSC. Patient recruitment was carried out by JHT, MM, JSD and DPS. The data was processed by JSD and MM, and was analysed by JSD with help from ACC, TWY and NMA. Laboratory sample processing and HPLC assays were performed by CJD and YRM. The manuscript was drafted by JSD and NMA. All authors had access to all data and contributed to the final draft of the paper. All authors read and approved the final manuscript.
